# Does Lithium Lower the Risk of Glaucoma—A Danish Nationwide Study

**DOI:** 10.1111/acps.70010

**Published:** 2025-07-03

**Authors:** Tomas Hajek, Orestes Forlenza, Marcelo Nicolela, Ann‐Eva Christensen, Henrik Vorum, René Ernst Nielsen

**Affiliations:** ^1^ Department of Psychiatry Dalhousie University Halifax Nova Scotia Canada; ^2^ Department of Psychiatry Faculty of Medicine, University of Sao Paulo Sao Paulo Brazil; ^3^ Department of Ophthalmology and Visual Sciences Dalhousie University Halifax Nova Scotia Canada; ^4^ Psychiatry Aalborg University Hospital Aalborg Denmark; ^5^ Aalborg University Hospital Aalborg Denmark; ^6^ Department of Clinical Medicine Aalborg University Aalborg Denmark

**Keywords:** bipolar disorders, glaucoma, lithium, pharmacoepidemiology

## Abstract

**Background:**

Glaucoma is an optic neuropathy caused by neurodegenerative loss of retinal ganglion cells (RGC). Mechanisms that contribute to RGC death include some of the same mechanisms involved in bipolar disorders (BD) and are the same mechanisms which are targeted by lithium (Li). We conducted a pharmacoepidemiological, population study in Denmark testing the links between Li prescriptions and risk of glaucoma.

**Study Design:**

A nationwide, register‐based historical, prospective cohort study of participants who were alive and over the age of 18 between January 1, 1996 and December 31, 2019. Participants were followed from the start of study until censoring or glaucoma.

**Study Results:**

A total of 7,683,398 individuals (51.3% females) contributing 121,366,461 person‐years were included in the study. In the general population, Li exposure was associated with developing glaucoma (HR = 1.10, 95% CI = 1.02–1.19, *p* = 0.01), but this association was not present in the population with BD (HR = 1.07, 95% CI = 0.93–1.22, *p* = 0.34). In the cumulative dosage analyses of the entire population, people with no Li prescription (HR = 0.78 95% CI = 0.66–0.93, *p* = 0.01) and between 365 defined daily doses (DDDs) and 5 × 365 DDDs of Li showed significantly reduced risk of glaucoma, relative to at least one prescription (HR = 0.79, 95% CI = 0.64–0.99, *p* < 0.05).

**Conclusion:**

BDs as indexed by Li prescription are associated with a greater risk of glaucoma. This is in keeping with generally increased rates of medical comorbidities in BD. While there was no clear dose–response relationship, some of the higher cumulative exposures to Li might be protective relative to a single prescription.

**Summary**

- Significant outcomes
  ○ Bipolar disorders, as indexed by Li treatment, were associated with greater risk of glaucoma
  ○ The risk of glaucoma was lower in people with some of the longer exposures to Li relative to those with a single prescription.
- Limitations
  ○ While this is not a randomized controlled trial, analyzing data from 7.7 million individuals and 121.4 million person‐years would not be feasible in an RCT.
  ○ Severity of glaucoma was not included in the population databases, so it is not clear if Li reduces the severity of glaucoma or the need for surgery.
  ○ We cannot differentiate whether the reduction of glaucoma risk with longer duration of treatment is related to neuroprotective effects of Li, healthier lifestyle, or closer medical follow‐ups in Li treated individuals.

## 1 Introduction

Glaucoma is an optic neuropathy caused by neurodegenerative loss of retinal ganglion cells (RGC). While increased intraocular pressure (IOP) is the most important risk factor for glaucoma, the neuropathy occurs frequently without IOP elevation, and most people with elevated pressure do not develop neuropathy [1]. Additionally, glaucoma may progress despite adequate IOP reduction [2]. Mechanisms that contribute to RGC death include oxidative stress, excitotoxicity, impaired mitochondrial energy metabolism, and withdrawal of trophic factors, leading to apoptosis. In this process, activation of intracellular calcium‐activated proteins (e.g., calcineurin) and calpain and proteases called “caspases,” increases expression of pro‐apoptotic genes (e.g., Bax/Bid) and down‐regulation of anti‐apoptotic genes (e.g., Bcl‐2/Bcl‐xl), which leads to programmed cell death [3, 4, 5, 6, 7].

Lithium (Li) is traditionally used in affective disorders [8]. Aside from mood‐stabilizing properties, it shows some of the most replicated and robust evidence for neuroprotective effects [9, 10, 11]. These have been documented on every level of investigation, including tissue cultures, animal models, human brain imaging studies [10, 11]. Pharmacoepidemiological studies showed an association between Li prescriptions or higher Li levels in drinking water and a lower risk of Alzheimer's dementia (AD) [12, 13, 14, 15]. A recent randomized, placebo‐controlled trial even demonstrated that long‐term, low‐dose Li treatment slowed cognitive‐functional decline and modified AD‐related biomarkers in older adults with mild cognitive impairment (MCI) [16, 17].

Mechanisms that contribute to RGC death include some of the same mechanisms involved in bipolar disorders (BD) and are the same mechanisms that are targeted by the neuroprotective effects of Li [18]. Specifically, Li acts against oxidative stress, reduces hyperexcitability of neurons, increases the levels of neuroprotective factors, and acts on the central component of the pro/antiapoptotic cascade—glycogen synthase kinase 3 beta (GSK3β). Through inhibition of GSK3B, it upregulates Bcl‐2, reduces levels of Bax, and has antiapoptotic mechanisms [9, 18].

Neuroprotective agents are needed as a complementary approach to glaucoma therapy. To date, only one large clinical trial utilizing a neuroprotective agent, that is, Memantine, has been reported in glaucoma, with no beneficial effects [19]. Preclinical studies have confirmed that Li modifies multiple mechanisms related to glaucoma pathophysiology. For example, through activation of the canonical Wnt pathway and β‐catenin, Li regulates the extracellular matrix expression in the trabecular meshwork and therefore facilitates aqueous outflow [3]. It supports both the survival and regeneration of RGC through a Bcl‐2‐dependent mechanism [4] and shows a cytoprotective effect on photoreceptor cells through activation of autophagy [5]. Inhibition of GSK3β by Li suppresses some of the biochemical effects of elevated pressure, and therefore protects against mitochondrial fission and cell death [6]. Last but not least, Li promotes neurite outgrowth, upregulates the transcriptional factor CREB, and improves the DNA repair pathway in primary cultured retinal neurocytes [7]. Even though the results of these studies were supportive, to the best of our knowledge, Li has not yet been studied as a prevention or treatment for glaucoma in human subjects.

## 2 Aims of the Study

If Li indeed helps RGC survival, we would expect to see lower rates of glaucoma in people on long‐term Li treatment. In this pharmacoepidemiological study, we investigated the association between prescriptions of Li and the risk of glaucoma on a population level with a large sample size and a very long duration of exposure.

## 3 Methods

### 3.1 Design

A nationwide, register‐based, historical, prospective cohort study.

### 3.2 Study Period

From January 1, 1996, to June 30, 2023. Participants were included from birth, contributing risk time from age 18 until the first occurrence of censoring, death, or glaucoma diagnosis.

### 3.3 Population

The cohort included individuals from the total Danish population alive and over 18 between January 1, 1996, and June 30, 2023. A subgroup was identified with a diagnosis of BD, characterized by the diagnosis of a single manic episode (F30.x) or BD (F31.x).

### 3.4 Explanatory Variables

#### 3.4.1 Li Exposure

Exposure was defined by a prescription for Li, Anatomical Therapeutic Chemical (ATC) code N05AN. Li treatment was initially defined as ever versus never exposure, and subsequently as a time‐varying variable in the Cox regression, with initially the number of prescriptions and later the number of defined daily doses (DDDs).

#### 3.4.2 Outcomes

Glaucoma was the primary outcome, defined by ICD‐10 H40.x diagnosis or glaucoma medication prescription (ATC codes: S01EA03, S01EA05, S01EC03, S01EC04, S01EC54, S01ED01, S01ED02, S01ED51, S01EE01, S01EE03‐05), whichever occurred first.

#### 3.4.3 Statistical Analyses

Descriptive statistics for sex distribution were presented as frequencies (percentage), while age at entry, age at glaucoma, and accumulated dosage were presented as median (25th percentile, 75th percentile). Cox proportional hazard regression assessed the association between Li exposure and glaucoma. Age was the underlying timescale. We present crude results and analyses adjusted for sex and age at entry, modelled using a restricted cubic spline with five knots placed at the percentiles [20].

We used three ways to quantify Li exposure—categorical (never versus ever), discrete quantitative variable (counting the actual number of DDDs), and lastly we categorized the number of DDDs into the following categories—no exposure, 1 prescription, +2 prescriptions, meaning a range of prescriptions between 2 and 364 DDDs, +365 DDDs, meaning a range of prescriptions between 365 DDDs and 1824 DDDs; and more than 5 × 365 DDDs. The single prescription is a reference group where we do not expect any effects of Li. The single prescription is used as a marker of the subtype of BD patients who are offered Li (but chose to discontinue) as compared to the broader whole BD group.

The primary model compared accumulated Li exposure (no Li, +1 prescription, +2 prescriptions, +365 DDDs, or +5 × 365 DDDs) adjusted for sex and age at entry, modelled as a restricted cubic spline, with +1 prescription as the reference, first in the whole sample and then in people with BD. Competing risk was addressed by censoring at death or immigration, with event frequencies presented. A *p* value < 0.05 was considered statistically significant. Analyses were conducted using Stata 18 [21] on the Danish Health Data Authority's server.

#### 3.4.4 Healthcare Registers Utilized

Data on psychiatric contacts and disorders were retrieved from the Danish Psychiatric Central Research Register (DPCRR), covering all psychiatric inpatient contacts from January 1, 1969, to December 31, 1995, and all in‐ and outpatient contacts in public psychiatric hospitals from January 1, 1996 [22].

Data on hospital contacts and somatic diseases were retrieved from the Danish National Patient Register (NPR), covering all somatic inpatient contacts from January 1, 1977, to December 31, 1995, and all in‐ and outpatient contacts in public hospitals from January 1, 1996 [23].

Data on deaths and immigration were retrieved from The Danish Civil Registration System, with population data available from April 2, 1968 [24].

Data on medication prescriptions are available from January 1, 1995, in the Danish National Prescription Registry (DNPR) [25]. In Denmark, all outpatient medications, such as Li or anti‐glaucoma medications, are purchased at pharmacies, and data are electronically recorded in the DNPR, including the patient's CPR number, ATC classification system code, dose, and number of tablets.

Register data can be linked to an individual via the unique personal identification number (CPR), assigned at birth or upon immigration [26].

## 4 Results

A total of 7,683,398 individuals (51.3% females) with a follow‐up period of 121,366,461 person‐years were included in the study. The median age at first Li prescription was approximately 46 years. For patients who developed glaucoma, the median age at diagnosis ranged from 65 to 71 years; see Tables 1 and 2 for more details.

**TABLE 1:** Sample description.

Columns by cumulative group	No lithium	+1 prescription	+2 prescriptions	+365 DDDs	+5 × 365 DDDs	Total
Sex, *n* (%)						
Female	3,410,503 (50.2)	16,972 (59.7)	14,352 (59.6)	8605 (59.1)	3329 (55.9)	3,453,761 (50.3)
Male	3,386,232 (49.8)	11,459 (40.3)	9742 (40.4)	5957 (40.9)	2624 (44.1)	3,416,014 (49.7)
Age at first glaucoma, median (iqi)	71 (60; 79)	67 (56; 76)	67 (56; 75)	67 (56; 74)	65 (56; 74)	71 (60; 79)
Age at entry in the group, median (iqi)	30 (18; 50)	46 (34; 58)	46 (34; 57)	47 (36; 58)	52 (41; 61)	30 (18; 50)
Accumulated dosage, median (iqi)	0 (0; 0)	25 (25; 34)	325 (175; 350)	1415 (725; 1800)	3320 (2430; 4793)	34 (0; 350)

**TABLE 2:** Crude rates of glaucoma in each exposure group.

	Whole population				Bipolar group			
Exposure group	Glaucoma or medical treatment for glaucoma	Death or immigration	Person‐years	Glaucoma/person years	Glaucoma or medical treatment for glaucoma	Death or immigration	Person‐years	Glaucoma/person years
No Li	203,452	2,016,057	121,366,461	0.00168	811	6621	659,281	0.00123
+1 prescription	130	1221	47,083	0.00276	39	329	14,387	0.00271
+2 prescriptions	230	2271	103,771	0.00222	81	931	48,106	0.00168
+365 DDDs	198	2115	95,248	0.00208	119	1224	60,373	0.00197
+5 × 365 DDDs	142	1015	50,662	0.00280	102	719	37,633	0.00271

In the crude analysis comparing ever versus never prescribed Li, ever being exposed to Li was significantly associated with developing glaucoma (HR = 1.12, 95% CI = 1.04–1.21, *p* < 0.001). This association remained significant after adjusting for sex and age (HR = 1.10, 95% CI = 1.02–1.19, *p* = 0.01). In a sub‐analysis restricted to patients with BD, Li exposure was not associated with glaucoma (HR = 1.07, 95% CI = 0.93–1.22, *p* = 0.34).

When quantifying Li exposure as a discrete quantitative variable, there was no significant association between the numbers of DDDs and glaucoma (HR = 1.00, 95% CI = 1.00–1.00, *p* = 0.99). When categorizing the numbers of DDDs, adjusting for sex and age at entry, and using +1 prescription as the reference, no Li exposure (HR = 0.78, 95% CI = 0.66–0.93, *p* = 0.01) and more than 365 DDDs were associated with a lower risk of glaucoma (HR = 0.79, 95% CI = 0.64–0.99, *p* < 0.05). Two or more prescriptions (HR = 0.89, 95% CI = 0.72–1.10, *p* = 0.29) and more than 5 × 365 DDDs (HR = 0.91, 95% CI = 0.72–1.16, *p* = 0.45) were not significantly associated with glaucoma risk; see Table 3.

**TABLE 3:** Hazard ratios for risk of glaucoma with +1 prescriptions as reference.

	HR	*p*	95% CI
Crude with continuous exposure			
Accumulated dosage	1.00	0.99	[1.00,1.00]
Crude whole population			
No Li	0.78	0.01	[0.66,0.93]
+1 prescription	1.00		
+2 prescriptions	0.89	0.29	[0.72,1.10]
+365 DDDs	0.79	0.04	[0.64,0.99]
+5 × 365 DDDs	0.90	0.39	[0.71,1.14]
Adjusted whole population			
No Li	0.77	0.00	[0.65,0.92]
+1 prescription	1.00		
+2 prescriptions	0.89	0.30	[0.72,1.11]
+365 DDDs	0.79	0.04	[0.64,0.99]
+5 × 365 DDDs	0.91	0.45	[0.72,1.16]
In the bipolar group			
No Li	0.59	0.00	[0.43,0.82]
+1 prescription	1.00		
+2 prescriptions	0.63	0.02	[0.43,0.93]
+365 DDDs	0.67	0.03	[0.46,0.96]
+5 × 365 DDDs	0.73	0.10	[0.51,1.06]

*Note:* Adjusted analysis for sex and age modelled as a restricted cubic spline with five knots.

In the sub analysis of individuals with BD, no Li (HR = 0.59, 95% CI = 0.43–0.82, *p* < 0.05), more than two prescriptions of Li (HR = 0.63, 95% CI = 0.43–0.93, *p* < 0.05) and more than 365 DDDs (HR = 0.67, 95% CI = 0.46–0.96, *p* < 0.05) were all associated with a lower risk of glaucoma. However, no significant association was found between more than 5 × 365 DDDs and glaucoma risk (HR = 0.73, 95% CI = 0.51–1.06, *p* = 0.1) in the analysis adjusted for sex and age, and with +1 prescription serving as the reference, see Table 3.

## 5 Discussion

In this study, individuals who ever purchased Li showed greater rates of glaucoma compared to those never exposed to Li, in both crude and adjusted analyses. This suggests the possibility of confounding by indication, as Li is primarily used to treat BD, which is associated with an increased risk of various medical conditions. Indeed, when analyses were restricted to people with BD, there was no difference in glaucoma risk between Li‐naïve and Li‐treated individuals. Next, we examined the association between glaucoma risk and different levels of Li exposure. Relative to those with at least one prescription of Li, no prescription and some of the longer exposures were associated with lower risk of glaucoma in either the full sample or the BD subgroup. At the same time, there was no clear dose–response relationship, as the group with the longest exposure to Li did not significantly differ from those with a single prescription.

The greater risk of glaucoma in people with a lifetime history of Li prescription (mostly BD individuals) relative to people who never purchased Li (mostly the general population without BD) was not seen when constraining the analyses to only people with BD. This is congruent with confounding by indication and is equivalent to another study, which showed a greater risk of dementia in Li‐treated participants when comparing to the general population, but actually a decreased or no difference in risk when comparing to other people with BD [15, 27]. Our results are also in keeping with other studies showing a greater risk of glaucoma in severe mental illness. Specifically, a previous population‐based study from Taiwan also demonstrated a significant association between the diagnosis of BD and glaucoma [28]. In another smaller study in veterans, those with serious mental illness (including BD) had a higher prevalence of glaucoma [29]. More generally, glaucoma has also been associated with other psychiatric disorders, including MDD, schizophrenia, and even anxiety disorders [29, 30, 31].

The increased risk of glaucoma in BD could be related to the use of medications potentially increasing IOP [32], better surveillance due to more frequent medical follow‐ups in Li‐treated individuals, or to higher rates of medical comorbidities in BD [18]. Medication use is the least likely explanation, as medications with the greatest propensity to increase IOP are rarely used in BD. We cannot rule this out completely, as a significant proportion of BD individuals, especially prior to diagnosis, are treated with antidepressants [33]. However, most of these medications would induce angle closure, which represents a small subset of all glaucoma. It is also possible that the increased rate of glaucoma in patients prescribed Li is biased by a greater frequency of medical follow‐ups in these individuals. This does not seem particularly likely, as studies, including those from Denmark, in fact show lower access/utilization of medical care in people with BD [34, 35]. However, it is possible that among BD individuals, those treated with Li have more regular medical check‐ups and may exhibit healthier behaviors than BD individuals on other medications. Lastly, people with BD show an increased risk of several medical comorbidities. While risk factors for glaucoma, beyond age, are not well established, it is possible that a greater burden of medical diseases would also result in a greater burden of glaucoma. In this regard, psychiatric medications could potentially contribute to the risk of glaucoma through increasing somatic comorbidities. Even more importantly, glaucoma is now considered a neurodegenerative disorder [1]. BD itself shows neuroprogression and is a risk factor for other neurodegenerative disorders, including dementia [36, 37]. Mechanisms involved in RGC loss, including downregulation of antiapoptotic genes, withdrawal of trophic factors, including BDNF, oxidative stress/mitochondrial dysfunction, excitotoxicity, and neuroinflammation are considered key drivers of the neuroprogressive changes in BD [38, 39, 40]. In keeping with this explanation, open‐angle glaucoma, which is more closely linked with neurodegeneration, was associated with most psychiatric disorders [28].

Any neuroprotective effects of medications, including Li, require an extended exposure. Therefore, the comparison of Li versus no Li, which does not consider the duration of exposure, is diluted by individuals with short‐term duration of Li treatment. Consequently, it is not surprising that within the BD group, the risk of glaucoma was comparable between the ever versus never Li‐treated individuals. Consequently, the analyses quantifying Li exposure are more informative. While showing that some of the higher cumulative exposures might be protective relative to a single prescription, these analyses did not provide a clear pattern of dose–response relationship, as outlined below.

Specifically, in the analyses of the entire population, we found that no exposure to Li was associated with a reduced risk of glaucoma (adjusted hazard ratio [HR] 0.77, 95% CI [0.65–0.92]) compared to at least one prescription, which served as the reference category. This could again represent confounding by indication and comparison between people without versus those with the diagnosis of BD. At the same time, the analyses constrained only to people with BD showed the same pattern, that is, no exposure to Li was associated with a reduced risk of glaucoma (adjusted HR = 0.59, 95% CI [0.43–0.82]) compared to at least one prescription. This cannot be explained by confounding by indication as even the individuals with no Li prescription had the diagnosis of BD. At the same time, this specific comparison carries the greatest risk of selection bias—that is, it includes Li naive BD individuals. There likely are systematic reasons why these individuals were never prescribed Li. These could include misdiagnosis of BD or very mild severity of illness, thus possibly conveying a lower risk of medical comorbidities, including glaucoma. At the same time, low compliance could be a reason not to prescribe Li and may indicate low compliance with other medical checkups or follow‐ups conveying a higher risk of medical comorbidities. In addition, as very few people are started on Li early in their illness, the non‐Li group had the lowest age at study entry and the longest follow‐up period. Consequently, the non‐Li group would have a high number of person years, most of them in young age, thus markedly lowering the glaucoma hazard in this group.

The comparisons between at least one and multiple Li prescriptions showed that repeated prescriptions of Li reduced the risk of glaucoma relative to at least one prescription. In the analyses of the entire population, a cumulative exposure totaling more than 365 DDDs but fewer than 5 × 365 DDDs (HR 0.79, 95% CI [0.64–0.99]) showed significantly reduced risk of glaucoma relative to at least one prescription. Among people with BD only, more than two and fewer than 5 × 365 DDDs were associated with a lower risk of glaucoma. The reduction of glaucoma risk in the 2–365 DDD group raises some issues. Even though the impact of Li on brain gray matter has been demonstrated already after 4 weeks of treatment [41], it is not likely that two prescriptions of Li could reduce the risk of glaucoma. One year of continuous Li prescriptions has been shown to be sufficient to reduce the risk of dementia in another study [42]. It is possible that the individuals who are prescribed Li for indications other than BD may stay on Li for shorter periods of time. So, the fact that the risk of glaucoma in the whole sample was not significantly lower in the group with two prescriptions to 365 DDDs may reflect that more people in this group had shorter/insufficient duration of exposure.

At the same time, there was not a clear dose–response relationship in these analyses, as the risk of glaucoma in people with the longest exposure (> 5 × 365 DDDs) was numerically, but not significantly lower from the risk associated with a at least one prescription of Li. This is not surprising, as the risk of glaucoma steeply increases with age, especially after age 60. The more than 5 × 365 DDDs group was the oldest group (age at entry 51 [41–60] years). The risk of glaucoma in this group should have been much higher than in the reference group of people with a at least one prescription (46 [34–58] years). Yet the rates of glaucoma were still numerically lower in this group than in the people with at least one prescription of Li. Even though we included age as the underlying time scale, as well as utilized cubic splines to adjust for a potentially non‐linear relationship with age, the generally low risk of glaucoma for most of the lifespan, which markedly increases at old age, is difficult to adjust for, especially as the age ranges between the reference group and the longest exposure groups are least overlapping. Nevertheless, even in analyses that did not control for age, the risk of glaucoma in this older group was numerically lower than the risk of glaucoma in the younger reference group. This is in keeping with another study in dementia, where the rate of dementia, which initially decreased in people treated with Li, remained only numerically, not significantly lower in the longest duration of exposure, highest age group [15].

The lower rates of glaucoma with repeated prescriptions of Li have several potential explanations. It is possible that this represents biochemical effects of Li on pathways that are involved in RGC death. Indeed, Li counteracts many of these mechanisms and shows promising effects in animal models of neurodegeneration [9]. Repeated prescriptions of Li also reduce the population risk of dementia, are associated with maintenance of GM and WM integrity in brain imaging studies [10] and people randomized to Li show better outcomes in other neurodegenerative disorders, including aMCI, AD, and ALS [12]. So, a direct effect of Li on neuronal survival and on neurodegenerative disorders is certainly conceivable.

The lower rates of glaucoma in Li treated individuals could also indicate a methodological artifact. Perhaps people with BD who show worse health may be more likely to be switched from Li or to never start it. Conversely, perhaps people who adhere to long‐term treatment with Li may generally be more likely to exhibit healthy and less likely to show unhealthy behaviors, including, for example, substance abuse [43]. Generally, aside from age, we do not know specific systemic risk or preventive factors for glaucoma. So, it is not clear what those behaviors increasing or decreasing the risk of glaucoma are and how prevalent they are in people who are prescribed Li. It is also possible that people on Li have fewer recurrences of BD, but there is no clear link between episodes of mood disorders and glaucoma risk. Also against these explanations is the fact that people on Li have lower mortality relative to individuals with BD who are prescribed other medications [44]. As age is the key risk factor for glaucoma, the risk of glaucoma should in fact be greater in the Li group, especially in those with long exposure. It is interesting that we see lower rates of glaucoma with longer prescriptions of Li, when regular check‐ups related to Li treatment could lead to neurological assessment and early identification of glaucoma, especially in people with longer duration of treatment. Furthermore, we selected the group of people with at least one prescription of Li as our reference to compare the longer exposures to people who were considered for Li treatment.

This study has the following limitations. While this is not a randomized controlled trial, analyzing data from 7.7 million individuals and 121.4 million person‐years would not be feasible in an RCT. As only diagnosis was included in the population databases, it is not clear if Li reduces the severity of glaucoma or the need for surgery. This could be important as a neuroprotective treatment might not prevent the development of the disease but might significantly alter the rate of progression/severity of that disease. Since we cannot condition on future events, the non‐Li group included those eventually prescribed Li. The intervals spent in each level of Li exposure were not identical, but we adjusted for this in the survival analyses. The association between glaucoma and age is not linear, so we controlled for age at entry modeled non‐linearly by a restricted cubic spline with five knots. Undiagnosed glaucoma cases, particularly in asymptomatic early stages, could have resulted in a limited number of outcomes and reduced statistical power to detect a potential true effect. Within the study, we do not believe the results could be a consequence of ascertainment bias, as current monitoring practices concerning Li treatment or treatment of severe mental disorders in general do not include examination for glaucoma.

## 6 Conclusion

This study provides indirect evidence for greater rates of glaucoma in BD, as documented by greater risk of glaucoma in Li treated individuals relative to the rest of the population, but not relative to people with BD not treated with Li. While the risk of glaucoma was reduced relative to at least one prescription in some of the longer exposure categories, there was no clear dose–response relationship, as the risk of glaucoma was lower in BD individuals with no Li prescription, but not in those with the longest Li exposure. These findings may reflect the complexities of glaucoma etiology, including skewed association with age, diverse pathophysiology, and frequent underdiagnosis, which increases the noise in the analyses. Despite these constraints, the study's large sample size and rigorous methodology provide a valuable foundation for future research. Further studies with targeted screening and detailed exposure assessments should focus on the association between Li and severity of glaucoma or the need for surgical interventions.

## Author Contributions

Tomas Hajek and Marcelo Nicolela conceived the study. All authors contributed to the design of the study. Ann‐Eva Christensen and René Ernst Nielsen collected the data. Ann‐Eva Christensen performed the analyses, with input from other authors, and summarized the results. Tomas Hajek wrote the manuscript with input from all other authors. All authors read and approved the final version.

## Conflicts of Interest

The authors declare no conflicts of interest.

## Peer Review

The peer review history for this article is available at https://www.webofscience.com/api/gateway/wos/peer‐review/10.1111/acps.70010.

## Data Availability

The data that support the findings of this study are not accessible to the public, and access is given after approval by The Danish Data Protection Agency and Statistics Denmark.
